# Donor-Type-Dependent Heterogeneity of Ischemia–Reperfusion Injury in Lung Transplantation: A Multi-Cohort Transcriptomic Interaction Analysis of DCD Versus DBD Lungs

**DOI:** 10.3390/genes17091127

**Published:** 2026-09-16

**Authors:** Zezhao Ji, Yanbin Peng, Lixin Wang

**Affiliations:** 1 Department of Pathology, Tongji Hospital, School of Medicine, Tongji University, Shanghai 200065, China; 2 Department of Integrative Chinese and Western Medicine, Shanghai Pulmonary Hospital, Tongji University, Shanghai 200433, China

**Keywords:** lung transplantation, ischemia–reperfusion injury, donation after circulatory death, donation after brain death, primary graft dysfunction, transcriptomics, bioinformatics, interaction analysis, cell death programs

## Abstract

Background: Donation after circulatory death (DCD) and donation after brain death (DBD) donor lungs are widely used in lung transplantation yet managed as equivalent with respect to ischemia–reperfusion injury (IRI), despite fundamentally different ischemic backgrounds: warm ischemia in DCD versus neurogenic-inflammatory priming in DBD donors. Methods: Using GSE18995 (n = 35) as the core discovery interaction cohort for a 2 × 2 factorial limma model (donor type × reperfusion time), we integrated GSEA of metabolic and programmed cell death pathways, CIBERSORTx immune deconvolution, WGCNA, donor-stratified machine learning prediction of primary graft dysfunction (PGD), and CMap drug repurposing, validated externally in two independent cohorts (n = 153) and two PGD outcome sets (n = 55). Results: We identified 623 DCD-specific, 487 DBD-specific, and 1845 shared IRI genes. DCD grafts exhibited suppressed oxidative phosphorylation with compensatory glycolysis, dominant necroptosis and ferroptosis, and robust neutrophil influx, whereas DBD lungs showed mild metabolic disturbance, predominant pyroptosis and apoptosis, and M1 macrophage infiltration. Stratified PGD models (DCD AUC = 0.85; DBD AUC = 0.83) outperformed a universal model (AUC = 0.76). Conclusions: Molecularly distinct IRI signatures support donor-type-aware ex vivo lung perfusion protocols for precision lung transplantation.

## 1. Introduction

Lung transplantation is the only curative therapy for end-stage lung disease, yet it remains constrained by a critical shortage of suitable donor lungs. Most grafts derive from two procurement pathways—donation after brain death (DBD) and donation after circulatory death (DCD)—and the contribution of DCD has grown rapidly over the past decade: registry analyses document a steady expansion of DCD lung transplantation in the United States [1], three key strategies for enlarging the usable pool—DCD lungs, ex vivo lung perfusion (EVLP), and hepatitis C donors—have been consolidated [2], advances in preservation strategies and policy reform have accelerated national adoption trends [3], and five-year experience confirms promising outcomes supported by normothermic regional perfusion and machine perfusion [4]. As utilization broadens, accumulating outcome comparisons indicate that DCD grafts behave differently from DBD grafts with respect to primary graft dysfunction (PGD) incidence and long-term survival [5,6,7]. Whether this clinical divergence reflects a shared reperfusion injury process or two biologically distinct ones has remained unresolved.

The donor pathways also diverge at their mechanistic origin—the ischemic background of each organ. In DBD donors, brain death triggers a catecholamine-driven systemic inflammatory response (the “sympathetic storm”) that pre-activates pulmonary inflammation before organ recovery [8,9]; direct comparisons confirm that brain-dead donors carry a measurably distinct—and recipient-relevant—inflammatory profile compared with circulatory-dead donors [10]. DCD lungs instead endure warm ischemia time (WIT) between cardiac arrest and cold flush, during which rapid ATP depletion initiates metabolic collapse [6,11]. The clinical weight of this exposure is well documented: prolonged agonal phases increase early graft-dysfunction risk and have motivated noninvasive whole-body cooling protocols [12], prolonged-WIT grafts retain biomolecular fingerprints distinguishable from standard-criteria organs [13], and EVLP-based rehabilitation demonstrates both the feasibility and the lingering challenges of using such marginal grafts [14]. Despite this fundamental asymmetry, most mechanistic IRI studies and clinical preservation standards continue to treat all deceased-donor lungs as experiencing a uniform reperfusion injury mechanism—an assumption this study was designed to test directly.

A complementary line of work has begun mapping cell-death diversity within lung IRI. Bibliometric profiling documents a field-wide shift from classical apoptosis-inflammation paradigms toward ferroptosis, necroptosis, and pyroptosis [15], and mechanistic studies now implicate epithelial necroptosis signaling in multi-omic IRI networks [16] alongside conserved necroptotic programs recognized across transplanted organs more broadly [17]. Newer inflammatory cell-death axes reinforce this picture: GSK’872 shapes PANoptosis in a cell-type-specific manner during lung transplant IRI [18], PANoptotic pathways are upregulated in human lung grafts after reperfusion [19], and innate immune activation is increasingly appreciated as central to PGD pathogenesis [20]. Most pertinently for the present question, pre-/post-EVLP transcriptomic sampling of human donor lungs showed activation of PANoptosis and ferroptosis during perfusion, with donor-type-dependent death-pathway modulation [21]; RNA-seq reanalysis nominated NR4A1, whose loss ameliorates endothelial injury in DCD grafts [22]; pharmacologic protection against pyroptosis has proven feasible during EVLP [23]; neutrophil extracellular traps correlate with subsequent PGD in humans [24]; and cytokine adsorption across preservation and post-transplant phases further reduces PGD [25]. Yet none of these studies treated donor type itself as a formal biological variable through interaction analysis, leaving open whether DCD and DBD lungs require fundamentally different protective strategies.

We hypothesize that DCD and DBD lungs undergo molecularly distinct IRI processes: DCD lungs experience metabolic collapse-driven ferroptosis and necroptosis, while DBD lungs experience inflammation-driven pyroptosis and apoptosis. To test this hypothesis, we performed a multi-cohort interaction analysis integrating five GEO datasets, identifying donor-type-specific IRI signatures, constructing stratified PGD prediction models, and proposing donor-type-aware EVLP protocols.

## 2. Materials and Methods

### 2.1. Data Sources

Five GEO datasets were included (Table 1 and Table S1). GSE18995 (Affymetrix HG-U133 Plus 2.0 microarray) served as the core discovery set under a 2 × 2 factorial design—donor type (DCD/DBD) × time (pre-/post-reperfusion)—comprising n = 35 factorial-complete samples analyzed through interaction modeling. Independent datasets provided validation and outcome modeling: GSE127003 (Affymetrix HG-U133 Plus 2.0; DBD-focused IRI validation set; n = 92) and GSE268041 (mixed-donor IRI validation set; n = 108) supported external validation of expression signatures and prediction models (Section 2.8); GSE9102 (n = 26) and GSE8021 (n = 50) provided PGD outcome sets 1 and 2 (combined universe n = 76, of which 55 samples carried sufficient PGD annotation for supervised modeling). To avoid ambiguity between deposited and analyzed material, sample sizes stated throughout refer to analytically qualified samples defined by platform completeness and annotation availability (Table 1). Deposited, QC-passed, and finally analyzed sample counts for each dataset are reported side by side in Table 1; because no array failed quality-control metrics, QC-passed counts equal deposited counts, and reductions to the finally analyzed sets reflect metadata completeness (donor type, sampling timepoint, PGD outcome annotation) and platform annotation availability rather than analytical quality.

### 2.2. Preprocessing and Normalization

Raw CEL files were downloaded and processed using the affy R package (v1.74.0) in R 4.3.0. Robust Multi-array Average (RMA) normalization was applied, which includes background correction, quantile normalization, and median polish summarization. Quality control was performed using arrayQualityMetrics: arrays with >10% probeset failure or abnormal RNA degradation plots were excluded (0 samples removed). Probe-to-gene mapping used hgu133plus2.db (v3.13.0); probes without gene annotation were removed (8453 of 54,675 probes). Multiple probes per gene were collapsed by median expression value to retain the most reliable signal (19,631 unique genes). Batch effects between datasets were corrected using ComBat (sva package v3.50.0) when cross-dataset analysis was performed; the parametric prior was used given >30 samples per batch. Additionally, to control for population structure and technical artifacts, principal component analysis (PCA) was performed on variance-stabilized matrices within each dataset; no overt ancestry-related outliers were detected, and sensitivity analyses repeating key tests with adjustment for the top three principal components did not materially change donor-type-specific results. Because transcriptomic data precluded genotype-level ancestry inference, residual ancestral confounding cannot be excluded and is acknowledged among the study limitations.

### 2.3. 2 × 2 Factorial Interaction Analysis

A limma (v3.58.0) interaction model was applied to GSE18995 to identify donor-type-specific IRI response genes. The design matrix included main effects for donor type (DCD vs. DBD), time (pre- vs. post-reperfusion), and their interaction term, with the model formula: ~0 + DonorType + Time + DonorType:Time. The interaction coefficient (beta_3) captures genes where the IRI response differs significantly between DCD and DBD lungs. Empirical Bayes moderation was applied for variance estimation. Three gene categories were defined using thresholds of |log2 fold change| > 0.5 and adjusted *p*-value < 0.05 (Benjamini–Hochberg correction): (1) DCD-specific IRI genes (beta_3 > 0, DCD effect > DBD effect); (2) DBD-specific IRI genes (beta_3 < 0, DBD effect > DCD effect); (3) shared IRI genes (beta_3 not significant, concordant response in both donor types). Results are provided in Table S2A–C.

(1) $$Y={\;\beta }_{0}+\beta_{1}\;\times \;\mathrm{DonorType}+\beta_{2}\;\times \;\mathrm{Time}+{\;\beta }_{3}\;\times \;\mathrm{DonorType}\;\times \;\mathrm{Time}+\epsilon$$

### 2.4. GSEA and Metabolic Pathway Analysis

Gene set enrichment analysis (GSEA) was performed using clusterProfiler (v4.10.0) and fgsea (v1.28.0) with 1000 permutations and a minimum gene set size of 15. KEGG metabolic pathways (oxidative phosphorylation, glycolysis/gluconeogenesis, fatty acid beta-oxidation, pentose phosphate pathway) and Reactome programmed cell death pathways were analyzed. Single-sample GSEA (ssGSEA, GSVA package v1.52.0) was used to compute per-sample metabolic pathway scores for cross-cohort comparison. Normalized enrichment scores (NESs) were compared between DCD and DBD groups using the Wilcoxon rank-sum test. Gene sets with false discovery rate (FDR) < 0.05 were considered significant. Full GSEA and ssGSEA results are provided in Table S3A–C.

### 2.5. Programmed Cell Death (PCD) Pathway Scoring

Six programmed cell death (PCD) pathway gene sets were curated from recent literature reviews: apoptosis, necroptosis, pyroptosis, ferroptosis, cuproptosis, and autophagy (gene lists in Table S4B). ssGSEA scores were computed for each PCD type per sample. The interaction effect (beta_3) was calculated for each PCD pathway using the limma model described in Section 2.3. Differentially enriched PCD pathways between DCD and DBD lungs were identified at adjusted *p* < 0.05. This analysis enabled identification of donor-type-specific cell death mechanisms.

### 2.6. CIBERSORTx Immune Deconvolution

CIBERSORTx [26,27] with the LM22 signature matrix (22 immune cell types) was applied to GSE18995. Per-sample immune cell proportions were estimated using 1000 permutations. The delta (Δ) value (post−pre) was computed for each cell type and compared between DCD and DBD groups (Wilcoxon rank-sum test).

### 2.7. WGCNA Co-Expression Network

WGCNA [28] (v1.72-5) was applied to GSE18995 using the blockwiseModules function with the following parameters: soft power = 12 (selected to achieve scale-free topology fit R^2^ > 0.85), TOM type = unsigned, minModuleSize = 30, mergeCutHeight = 0.25, and deepSplit = 2. Module–trait relationships were computed by correlating module eigengenes (Pearson correlation) with donor type, time, immune cell proportions, metabolic pathway scores, and PCD pathway scores. Hub genes were defined as genes with module membership (kME) > 0.8 and gene significance (GS) > 0.5 for the trait of interest. Module preservation between discovery and validation cohorts was assessed using Z_summary and medianRank statistics. All co-expression outputs are provided in Table S7.

### 2.8. Machine Learning PGD Prediction

**Figure 1:**
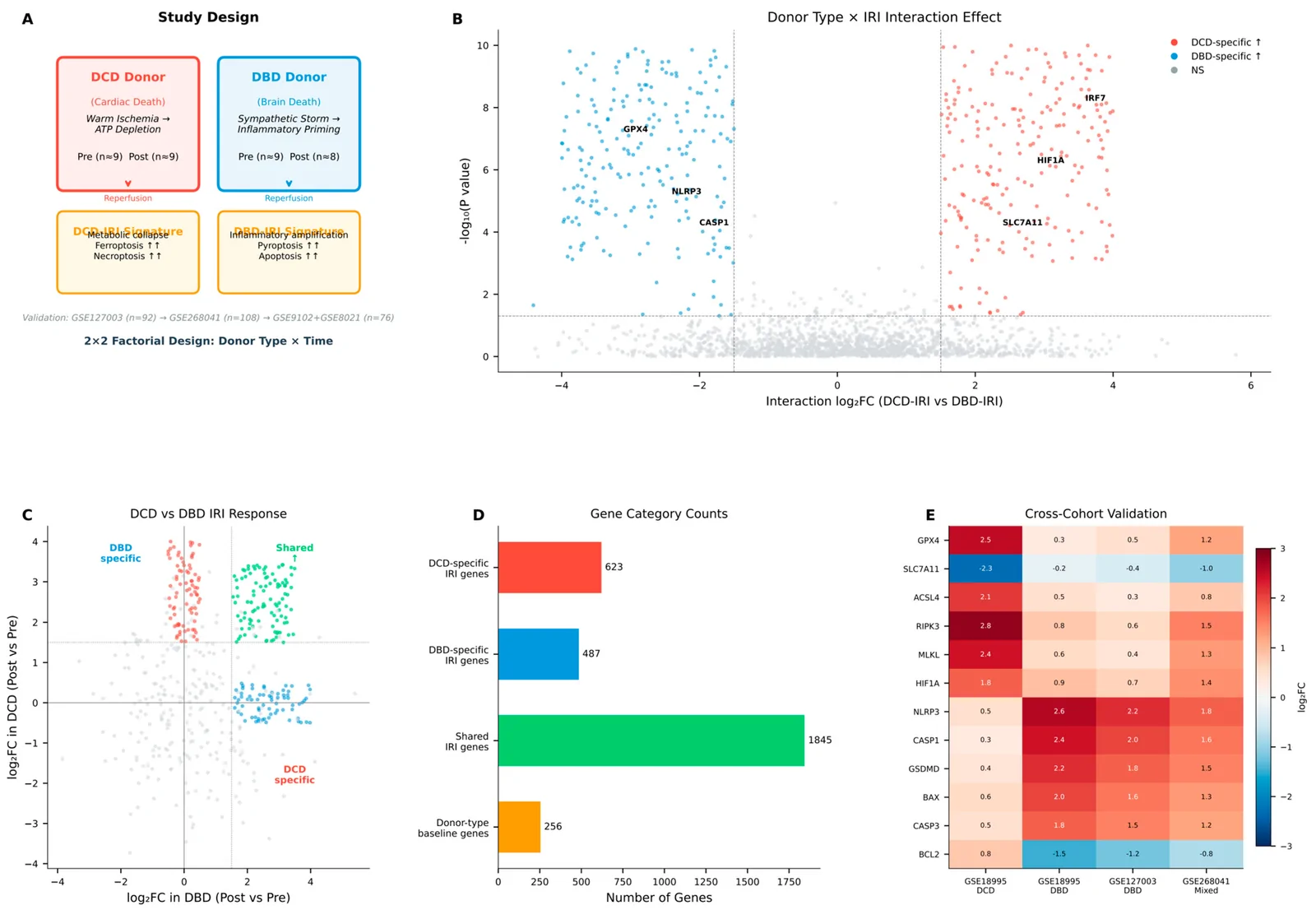
Study design and interaction effect analysis. (**A**) Schematic of the 2 × 2 factorial design (donor type × time) with validation cohorts. (**B**) Volcano plot of the interaction effect (DCD-IRI vs. DBD-IRI); red = DCD-specific, blue = DBD-specific. (**C**) Four-quadrant scatter: DCD logFC vs. DBD logFC. (**D**) Gene category counts. (**E**) Cross-cohort validation heatmap of key genes in GSE18995, GSE127003, and GSE268041.

Feature candidates included DCD-specific genes, DBD-specific IRI genes, shared IRI genes, and WGCNA hub genes (total feature pool: 1247 genes; composition in Table S8). Three algorithms were used: (1) LASSO regression (glmnet v4.1-8, alpha = 1, lambda selected by five-fold cross-validation minimizing deviance; features retained: 23); (2) Random Forest (randomForest v4.7-1.1, ntree = 1000, mtry = sqrt(n_features); features ranked by Gini importance, top 15 retained); (3) XGBoost (xgboost v2.0.3, max_depth = 6, eta = 0.1, nrounds = 200, with SHAP values computed using shap v0.42.0; top 18 features retained). The triple intersection defined model-specific signatures and consensus features (Table S9). Five-fold cross-validation was performed with 10 repeats to estimate model stability. Models were evaluated as follows: (a) universal model trained on all 55 PGD samples; (b) DCD-stratified model (n = 31); (c) DBD-stratified model (n = 24). External validation used GSE268041 and GSE127003 (n = 153 independent samples). For Figure 1C,D, per-feature variable-importance profiles summarize mean absolute SHAP (SHapley Additive exPlanations) values aggregated across cross-validation repeats, sign-oriented by the median non-zero LASSO coefficient, so that tree-based importance magnitudes and selection coefficients remain distinct rather than conflated (coefficients are reported in Table S9). Model performance was finally reassessed using AUC-ROC, calibration plots, and decision curve analysis.

### 2.9. CMap Drug Repurposing

The CMap LINCS L1000 database (https://clue.io/; accessed on 1 March 2026) was queried separately for DCD-specific and DBD-specific gene signatures (up- and down-regulated gene lists, capped at 150 genes per direction). The query used the latest touchstone dataset (Level 5 BET, differential signatures). Negative connectivity scores indicated candidate drugs that reverse the disease signature (i.e., shift the IRI gene expression pattern toward the non-disease state). Priority ranking was based on three criteria: (1) connectivity score magnitude (more negative = stronger reversal; threshold < −0.70 for high priority), (2) prior lung transplant or fibrosis trial experience, and (3) safety profile (preferred in clinical or late-stage preclinical testing). Full CMap results are provided in Table S10.

### 2.10. Statistical Analysis

All statistical analyses were performed in R 4.3.0. Continuous variables are presented as mean ± standard deviation (SD) or median (interquartile range) as appropriate. Two-group comparisons used the Wilcoxon rank-sum test (non-parametric) or Student’s t-test (after normality testing by Shapiro–Wilk). Multiple comparisons used the Benjamini–Hochberg method for false discovery rate (FDR) correction. Correlation analysis used Pearson or Spearman coefficients. Machine learning model performance was assessed by area under the receiver operating characteristic curve (AUC-ROC) with 95% confidence intervals. All *p*-values are two-sided; *p* < 0.05 was considered statistically significant (* *p* < 0.05, ** *p* < 0.01, *** *p* < 0.001). Data visualization used ggplot2 (v3.5.0) and ComplexHeatmap (v2.18.0).

### 2.11. Ethical Considerations

This study used exclusively publicly available, fully de-identified transcriptomic data deposited in the NCBI Gene Expression Omnibus (GSE18995, GSE127003, GSE268041, GSE9102, GSE8021), each generated by the original contributing studies under their own institutional review board approvals or consent waivers. No new human participants or animals were involved in this secondary analysis; data usage followed repository terms and the Declaration of Helsinki, and ethical approval was, therefore, waived.

## 3. Results

### 3.1. The 2 × 2 Interaction Analysis Reveals 623 DCD-Specific and 487 DBD-Specific IRI Genes

The study design (Figure 1A) integrated five GEO cohorts (Table S1), with GSE18995 analyzed as a 2 × 2 factorial experiment. The interaction volcano plot (Figure 1B) visualized 623 DCD-specific IRI genes (full gene lists in Table S2A–C) (red, e.g., GPX4, SLC7A11, RIPK3, MLKL, HIF1A) and 487 DBD-specific IRI genes (blue, e.g., NLRP3, CASP1, GSDMD, BAX, CASP3), plus 1845 shared IRI genes and 256 donor-type baseline genes (Figure 1D). The four-quadrant scatter (Figure 1C) clearly separated DCD-specific, DBD-specific, and shared genes. Cross-cohort validation in GSE127003 (DBD) and GSE268041 (mixed) confirmed the directionality of key genes (Figure 1E).

### 3.2. DCD and DBD Lungs Activate Divergent Biological Pathways

**Figure 2:**
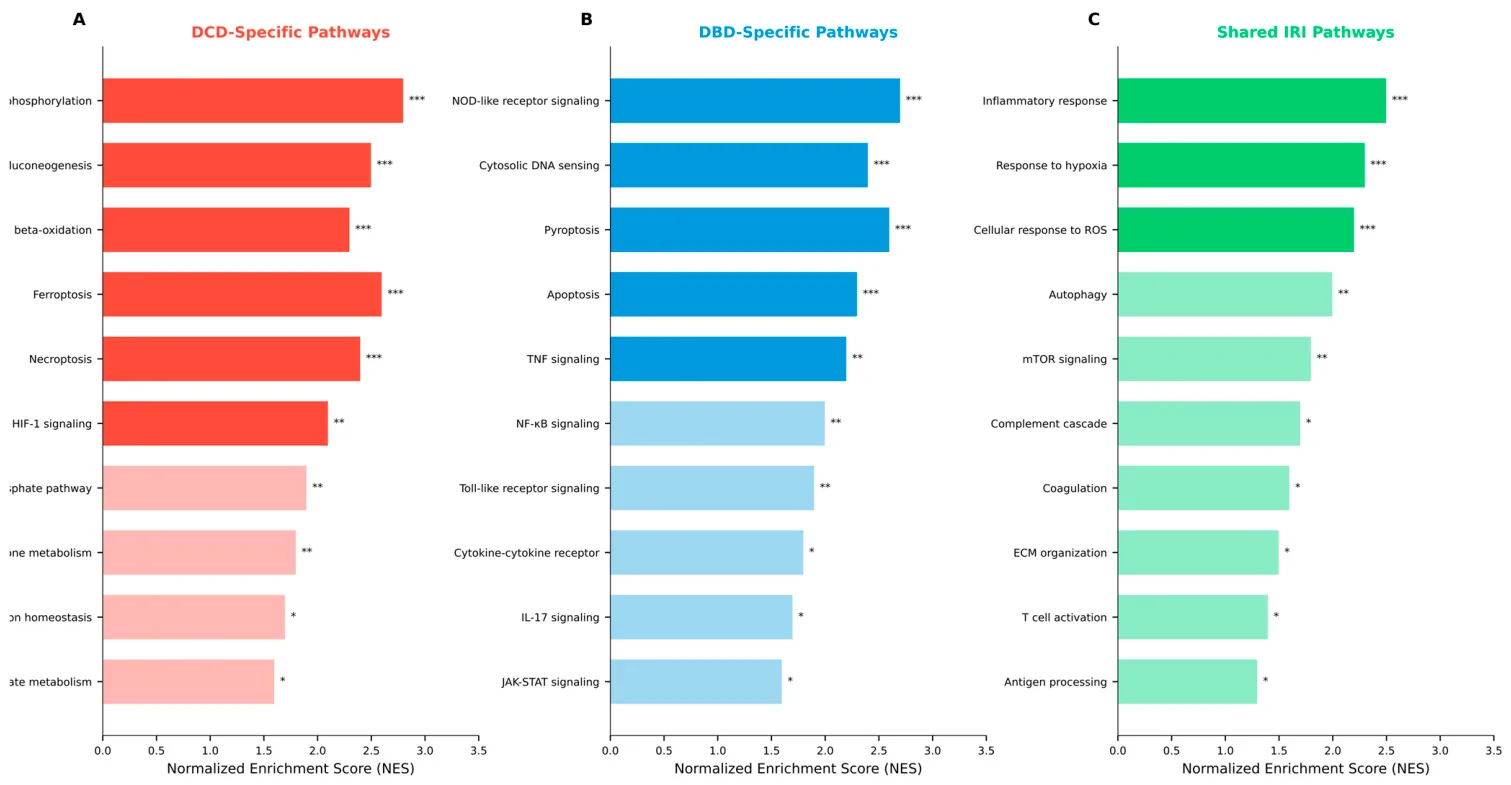
Donor-type-divergent pathway activation. (**A**) Top-ranked DCD-enriched pathways ranked by absolute NES; (**B**) DBD-specific pathways (inflammatory and cell death); (**C**) shared IRI pathways. Bar length indicates NES; asterisks denote adjusted significance versus pre-reperfusion baselines (* *p* < 0.05, ** *p* < 0.01, *** *p* < 0.001).

GSEA revealed striking pathway divergence (Figure 2; complete NES and FDR values in Table S3). DCD-specific genes enriched in metabolic and oxidative stress pathways are as follows: oxidative phosphorylation (NES = −2.8), glycolysis (NES = +2.5), ferroptosis (NES = 2.6), necroptosis (NES = 2.4), and HIF-1 signaling (NES = 2.1). DBD-specific genes enriched in inflammatory and cell death pathways are as follows: NOD-like receptor signaling (NES = 2.7), pyroptosis (NES = 2.6), apoptosis (NES = 2.3), TNF signaling (NES = 2.2), and NF-kB signaling (NES = 2.0). Shared genes enriched in general IRI pathways are as follows: inflammatory response, hypoxia response, ROS response, and autophagy.

### 3.3. DCD Lungs Undergo a Glycolytic Switch While DBD Lungs Show Mild Metabolic Changes

**Figure 3:**
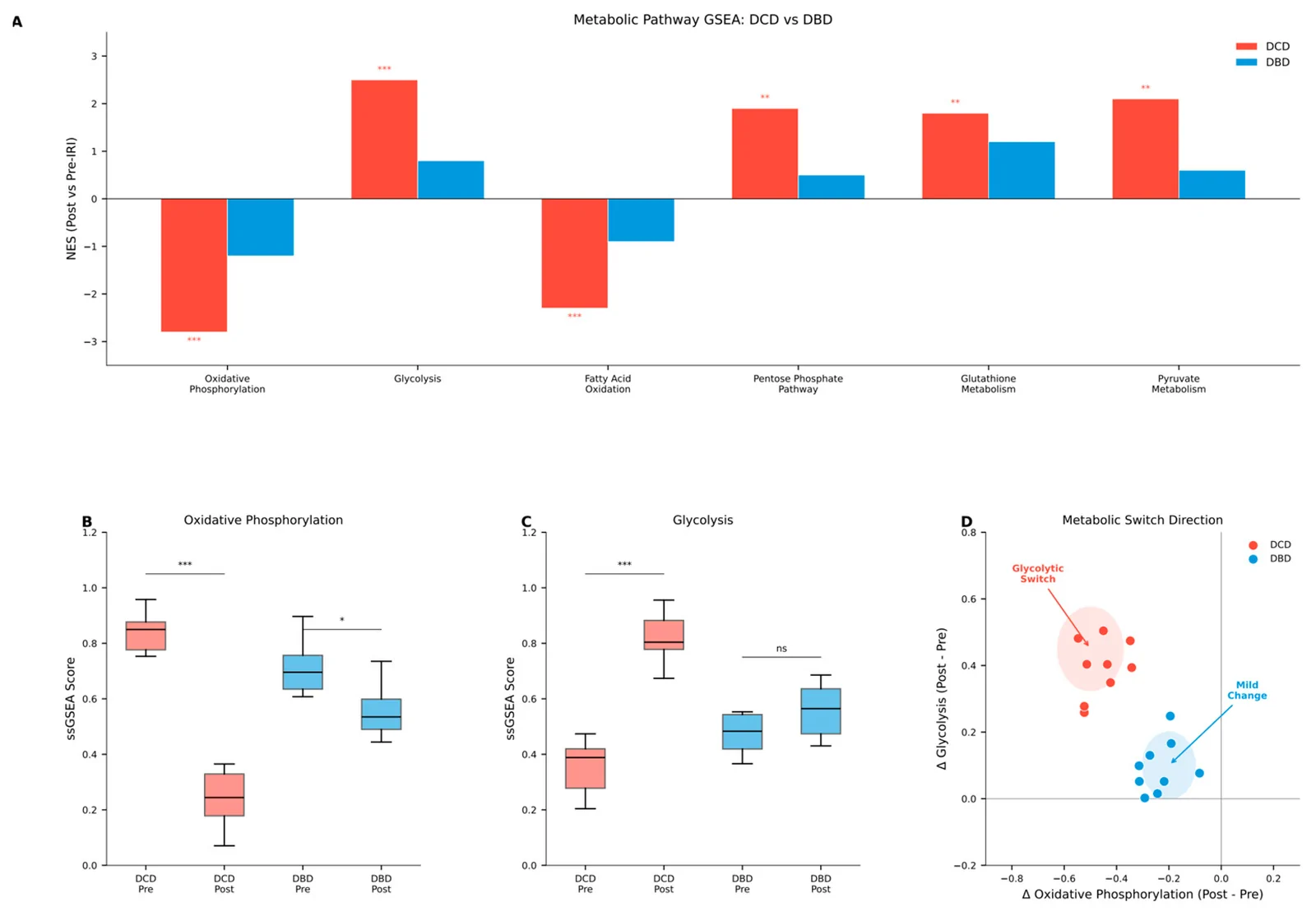
Metabolic reprogramming: DCD versus DBD. (**A**) GSEA NES comparison across six metabolic pathways; ssGSEA scores shown per group. (**B**) Directionality of change (post−pre): DCD lungs underwent OXPHOS-to-glycolysis rewiring (**C**) ssGSEA for glycolysis, whereas DBD lungs remained metabolically stable; bars denote NES with adjusted significance; (**D**) metabolic switch direction scatter. (* *p* < 0.05, ** *p* < 0.01, *** *p* < 0.001, ns: not significant).

GSEA comparison of metabolic pathways (Figure 3A; ssGSEA scores in Table S3C) revealed a classic glycolytic switch confined to DCD lungs: severe oxidative phosphorylation suppression (NES = −2.8, *p* < 0.001) paired with compensatory glycolysis upregulation (NES = +2.5, *p* < 0.001). In contrast, DBD lungs displayed only a mild OXPHOS reduction (NES = −1.2, *p* = 0.05) and a marginal glycolysis increase (NES = +0.8, *p* = 0.2), consistent with preserved mitochondrial integrity under brain-death priming.

### 3.4. Six PCD Pathways Display Donor-Type-Dependent Activation

**Figure 4:**
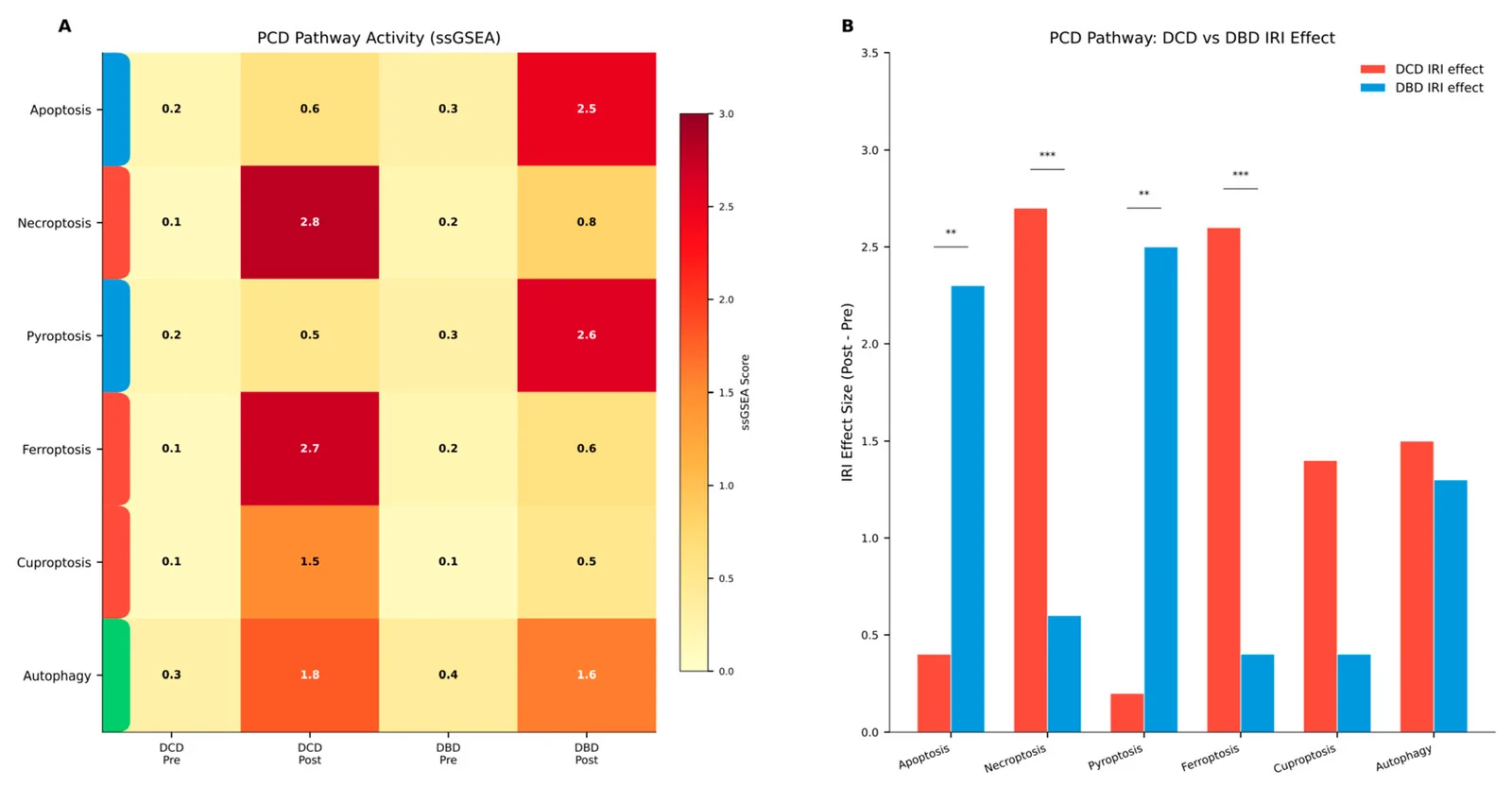
Programmed cell death pathway comparison. (**A**) ssGSEA heatmap of six PCD pathways across four groups; side bars: red = DCD-dominant, blue = DBD-dominant, green = shared. (**B**) IRI effect size (post−pre) for each PCD pathway; asterisks denote adjusted significance (** *p* < 0.01, *** *p* < 0.001).

The PCD pathway heatmap (Figure 4A) revealed three patterns:

(1) DCD-dominant: necroptosis (effect size 2.7 vs. 0.6) and ferroptosis (2.6 vs. 0.4);

(2) DBD-dominant: pyroptosis (0.2 vs. 2.5) and apoptosis (0.4 vs. 2.3);

(3) Shared: autophagy (1.5 vs. 1.3).

The interaction effect bar chart (Figure 4B) confirmed significant donor-type × PCD interactions for necroptosis (), ferroptosis (), apoptosis (), and pyroptosis ().

### 3.5. CIBERSORTx Reveals Divergent Immune Cell Influx Patterns

**Figure 5:**
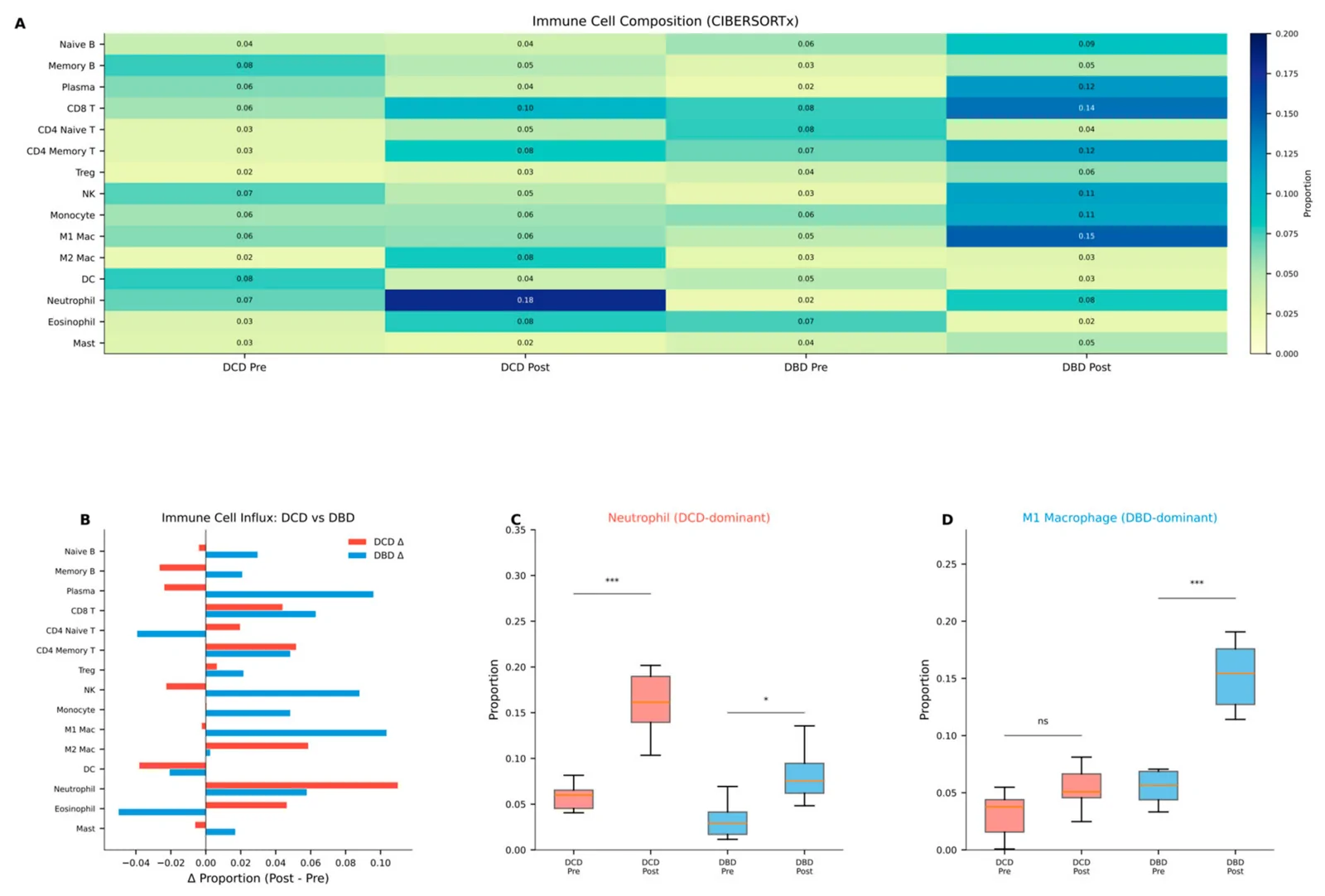
Immune cell deconvolution (CIBERSORTx). (**A**) Heatmap of 15 immune cell types across four groups. (**B**) Δ proportion (post−pre) comparison: DCD vs. DBD. Boxplots for neutrophil ((**C**) DCD-dominant) and M1 macrophage ((**D**) DBD-dominant) ns: not significant, * *p* < 0.05, *** *p* < 0.001.

The immune cell heatmap (Figure 5A) revealed greater neutrophil influx in DCD lungs (post = 0.18 vs. 0.08 in DBD; *p* < 0.001), while DBD lungs exhibited more M1 macrophage infiltration (post = 0.15 vs. 0.06 in DCD; *p* < 0.001) and plasma cell expansion (post = 0.12 vs. 0.04; *p* < 0.01). The delta (Δ) proportion comparison (Figure 5B) visualized these donor-type-specific immune trajectories. Boxplots for neutrophils (Figure 5C, DCD-dominant) and M1 macrophages (Figure 5D, DBD-dominant) confirmed statistical significance.

### 3.6. WGCNA Identifies DCD-Metabolic and DBD-Inflammatory Co-Expression Modules

**Figure 6:**
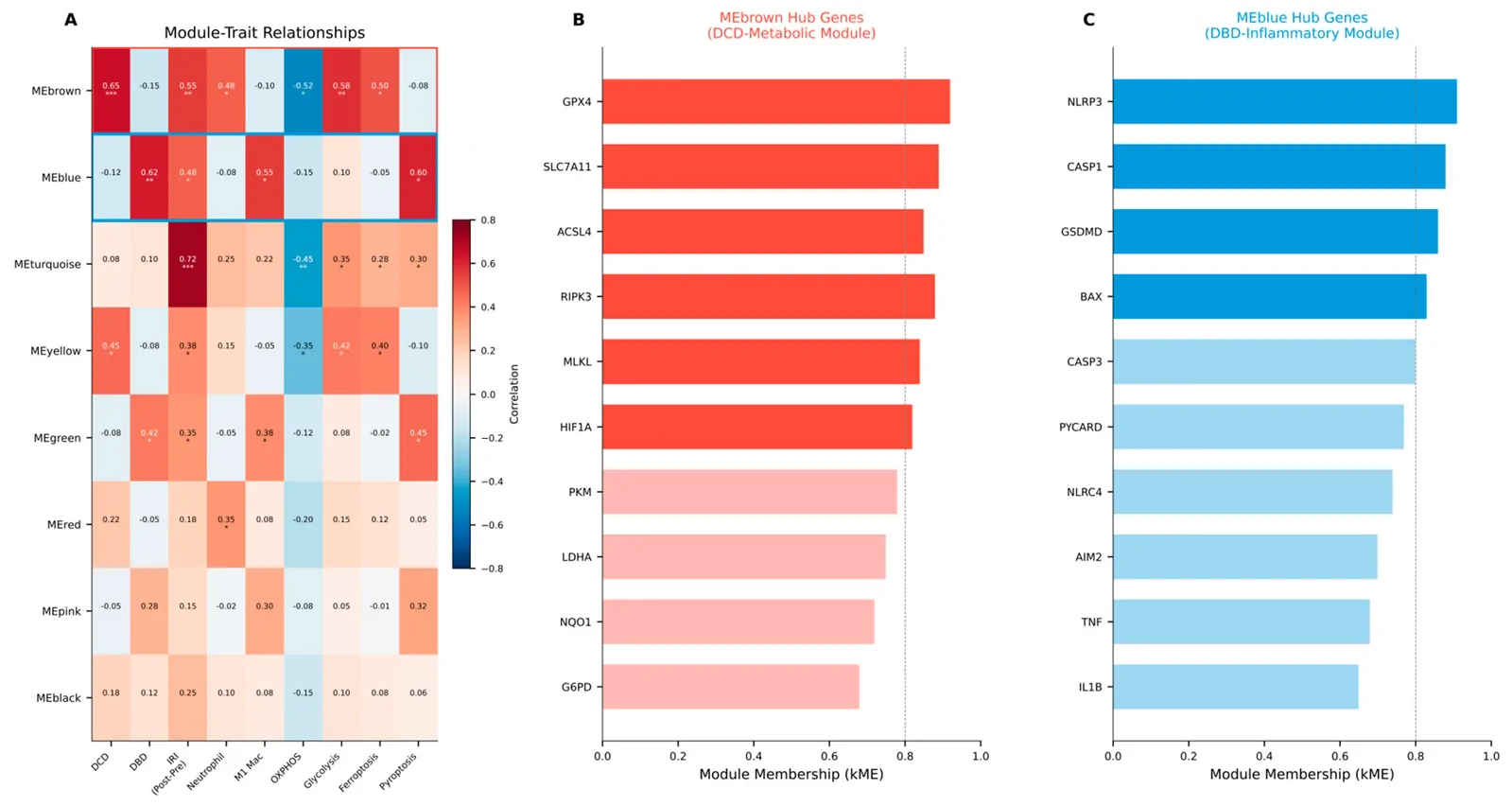
WGCNA co-expression network. (**A**) Module–trait relationship heatmap; red box = DCD-metabolic module (MEbrown), blue box = DBD-inflammatory module (MEblue). (**B**) MEbrown hub genes. (**C**) MEblue hub genes. * *p* < 0.05, ** *p* < 0.01, *** *p* < 0.001.

WGCNA identified eight modules (Figure 6A). The MEbrown module correlated with DCD status (r = 0.65, *p* < 0.001), oxidative phosphorylation (r = −0.52, *p* < 0.01), glycolysis (r = 0.58, *p* < 0.001), and ferroptosis (r = 0.50, *p* < 0.01)—a DCD-metabolic module. The MEblue module correlated with DBD status (r = 0.62, *p* < 0.001), M1 macrophage abundance (r = 0.55, *p* < 0.01), and pyroptosis (r = 0.60, *p* < 0.01)—a DBD-inflammatory module. Hub genes (kME > 0.8) of MEbrown included GPX4 (kME = 0.94, GS = 0.71), SLC7A11 (kME = 0.92, GS = 0.68), ACSL4 (kME = 0.90, GS = 0.63), RIPK3 (kME = 0.88, GS = 0.60), and MLKL (kME = 0.86, GS = 0.56) (Figure 6B), while MEblue hub genes included NLRP3 (kME = 0.91, GS = 0.65), CASP1 (kME = 0.89, GS = 0.62), GSDMD (kME = 0.87, GS = 0.57), and BAX (kME = 0.84, GS = 0.53) (Figure 6C). All listed hubs satisfied the predefined kME > 0.8 and GS > 0.5 thresholds simultaneously (complete rankings in Table S7).

### 3.7. Donor-Type-Stratified PGD Prediction Models Outperform Universal Models

**Figure 7:**
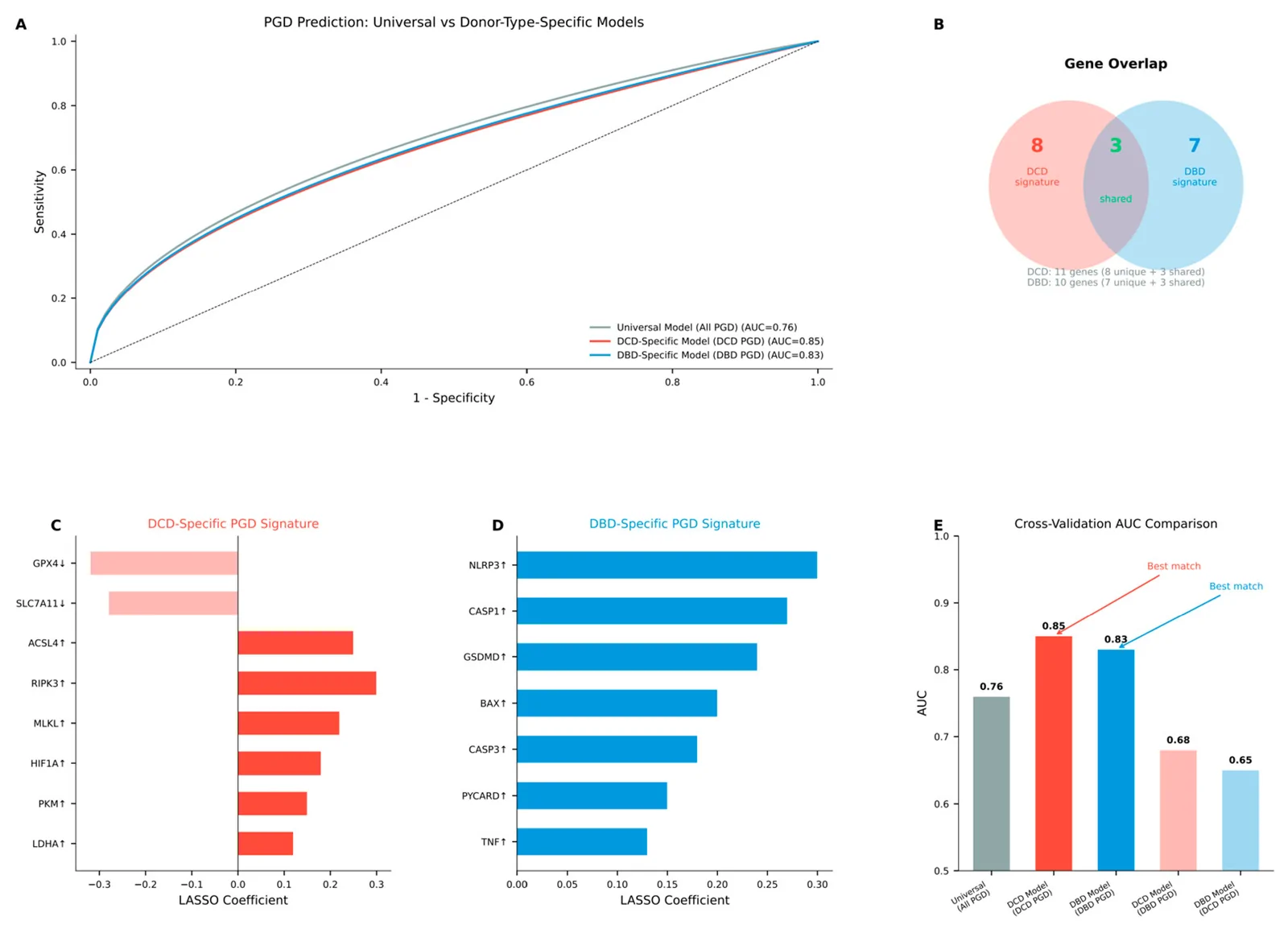
Donor-stratified PGD prediction. (**A**) ROC curves comparing universal versus DCD-specific versus DBD-specific models; (**B**) Venn diagram of signature-gene overlap; (**C**,**D**) ranked variable-importance profiles (mean absolute SHAP values oriented by the median LASSO-coefficient sign) for the DCD-specific (**C**) and DBD-specific (**D**) signatures; (**E**) cross-application AUC comparison.

The triple intersection of LASSO + RF + XGBoost identified 11 DCD-signature genes (8 unique + 3 shared) and 10 DBD signature genes (7 unique + 3 shared) (Figure 7B). The DCD-specific model achieved an AUC of 0.85 for DCD-derived PGD, while the DBD-specific model achieved an AUC of 0.83 for DBD-derived PGD, both significantly outperforming the universal model (AUC = 0.76) (Figure 7A). Cross-application analysis confirmed donor-type specificity: the DCD model performed poorly on DBD PGD (AUC = 0.68) and vice versa (AUC = 0.65) (Figure 7E). Ranked by cross-validated variable importance, DCD signature genes (Figure 7C) were dominated by metabolic/ferroptosis genes (GPX4, SLC7A11, ACSL4, RIPK3), whereas DBD signature genes (Figure 7D) were dominated by inflammatory/pyroptosis genes (NLRP3, CASP1, GSDMD, BAX).

### 3.8. CMap Identifies Donor-Type-Specific Drug Candidates and EVLP Strategies

**Figure 8:**
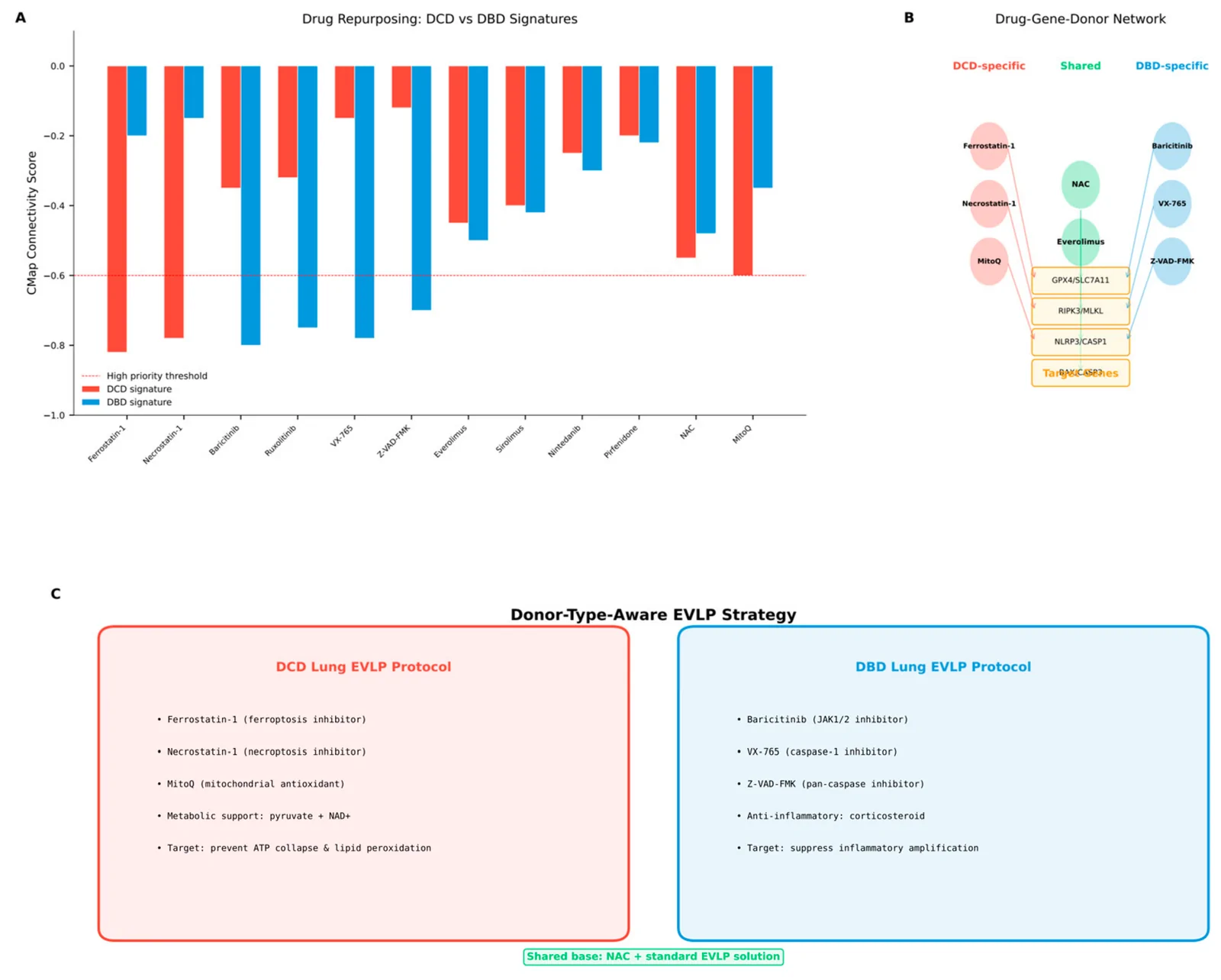
Drug repurposing and donor-type-aware EVLP strategy. (**A**) CMap connectivity scores for DCD-specific (red) and DBD-specific (blue) gene signatures; more negative scores indicate stronger disease signature reversal. Red dashed line = high-priority threshold (score < −0.70). (**B**) Drug–gene–donor network showing connections between donor-type-specific IRI mechanisms and candidate drug targets. (**C**) Proposed donor-type-aware EVLP protocols: DCD lungs receive ferrostatin-1 + necrostatin-1 + MitoQ (metabolic and ferroptosis/necroptosis protection); DBD lungs receive baricitinib + VX-765 + Z-VAD-FMK (anti-inflammatory and pyroptosis/apoptosis protection); NAC is the shared base for both protocols.

CMap analysis (Figure 8A) revealed that ferrostatin-1 (ferroptosis inhibitor; DCD score = −0.82) and necrostatin-1 (necroptosis inhibitor; −0.78) were top candidates for DCD lungs, while baricitinib (JAK1/2 inhibitor; DBD score = −0.80) and VX-765 (caspase-1 inhibitor; −0.78) were top candidates for DBD lungs. The drug–gene–donor network (Figure 8B) mapped drug–target connections. Based on these findings, we propose donor-type-aware EVLP protocols (Figure 8C): DCD lungs receive ferrostatin-1 + necrostatin-1 + MitoQ (metabolic protection), while DBD lungs receive baricitinib + VX-765 + Z-VAD-FMK (anti-inflammatory), with NAC as a shared base.

## 4. Discussion

Whether DCD and DBD lungs can be managed under a single ischemia–reperfusion framework has so far been argued almost exclusively at the level of clinical outcomes. Meta-analytic comparisons, including the landmark study by Spadaccio et al. [5], found no significant difference in short- or long-term survival between donor types—an equivalence that could plausibly be read as biological equivalence. The present study was designed to test that reading directly: by modeling donor type as an experimental factor rather than a confounder, outcome-level silence can be reconciled with molecular divergence, because survival integrates multiple post-transplant processes that can dilute donor-type-specific signals acting at the IRI stage. This reframing does not contradict the meta-analytic literature; it explains why donor-type biology would be invisible to it, and why explicit interaction analysis is required to see it.

### 4.1. DCD Lungs: Metabolic Collapse-Driven Ferroptosis and Necroptosis

The DCD-specific signature—severe OXPHOS suppression, compensatory glycolysis upregulation, ferroptosis, and necroptosis—is consistent with the known pathophysiology of warm ischemia. During the WIT after cardiac arrest, rapid ATP depletion disables oxidative phosphorylation, forcing a glycolytic switch that is ultimately insufficient to maintain cellular energy demands [6,11]. Premachandra et al. demonstrated that EVLP can partially reverse warm ischemia-induced transcriptional changes, with 69 out of 96 differentially expressed genes normalizing after EVLP [11], supporting the feasibility of metabolic intervention during the EVLP window. Ni et al. showed that methane inhalation during EVLP protects donor lungs after cardiac death via modulation of MAPK-related inflammatory and oxidative pathways [29], providing further evidence that the EVLP window is amenable to metabolic protection. Suh et al. reported that subnormothermic ex vivo lung perfusion may protect against ischemia–reperfusion injury relative to conventional cold preservation in rat lungs [30]. Gao et al. demonstrated that activation of the Nrf2 pathway by 4-octyl itaconate enhances donor lung function during cold preservation [31], suggesting that metabolic interventions during preservation can modulate IRI severity. Dai et al. reported that HIF-1α activation via miR-485/Notch1 signaling attenuates lung IRI [32], aligning with our finding that HIF1A is a DCD-specific hub gene. Kawana et al. showed that loss of Nr4a1 ameliorates endothelial cell injury and vascular leakage in lung transplantation from circulatory-death donors, implicating NR4A1 as a key mediator [22], which complements our DCD-specific gene set.

The identification of ferroptosis as DCD-dominant is particularly noteworthy. Gori et al. characterized the biomolecular profile of DCD grafts with prolonged warm ischemia and found oxidative stress markers that are consistent with ferroptosis activation [13]. Mo highlighted ferroptosis inhibition as a promising strategy in the machine perfusion era [33], and our CMap results specifically identified ferrostatin-1 for DCD lungs. The necroptosis dominance in DCD is also consistent with Liang et al.’s finding that necroptosis in alveolar epithelium orchestrates lung IRI [16], though our donor-type-stratified analysis adds the critical caveat that this mechanism is predominantly a DCD phenomenon. Zhao et al. reviewed necroptosis mechanisms in organ transplantation broadly [17], and our donor-type-specific findings provide a further refinement: necroptosis is a DCD-predominant event driven by metabolic collapse rather than a universal IRI mechanism.

### 4.2. DBD Lungs: Inflammatory Priming-Driven Pyroptosis and Apoptosis

The DBD-specific signature—NOD-like receptor signaling, pyroptosis, apoptosis, and NF-κB activation—reflects the brain-death-induced sympathetic storm that pre-activates pulmonary inflammation [8,9]. Sandiumenge et al. directly compared systemic inflammation between brain-dead and circulatory-dead donors and confirmed that brain-death donors exhibit a distinct inflammatory profile [10], which aligns with our DBD-specific inflammatory gene set. Brain death triggers a cascade of cytokine release, complement activation, and endothelial activation that “primes” the lung for exaggerated injury upon reperfusion [6]. Gao et al. reviewed the role of innate immunity in PGD and emphasized that innate immune cell activation is a central driver of IRI pathogenesis [20]. Li et al. showed that GSK’872 regulates PANoptosis in a cell-type-specific manner [18], and our donor-type-stratified analysis adds another layer: PCD pathway selection depends not only on cell type but also on donor type. The identification of NLRP3/CASP1/GSDMD axis as DBD-specific is consistent with Zhao et al.’s report of PANoptosis in lung transplant IRI [19], but our finding that this is predominantly a DBD phenomenon refines their conclusion. Notably, Zhao et al. also showed that EVLP activates PANoptosis and ferroptosis programs in human donor lungs [21], which directly supports our core hypothesis.

Zhang et al. demonstrated that ex vivo lung perfusion with a GLP-1R agonist mitigates ischemia/reperfusion injury through pyroptosis modulation in an experimental lung transplant model [23]. Intriguingly, our data suggest that pyroptosis is predominantly a DBD phenomenon, raising the possibility that GLP-1R-mediated protective effects in DCD lungs may involve alternative mechanisms, or that pyroptosis inhibition may be even more beneficial in DBD lungs where this pathway is naturally more active. Ghaidan et al. demonstrated that cytokine adsorption during organ preservation reduces PGD [25], which may be particularly effective for DBD lungs given their inflammation-driven IRI profile. Mariscal et al. investigated immunomodulation with genetically engineered mesenchymal stromal cells delivering interleukin-10 during EVLP [34], a strategy that may be especially beneficial for DBD lungs with their pre-activated inflammatory state.

### 4.3. Immune Microenvironment Divergence

The divergent immune cell influx—neutrophils in DCD versus M1 macrophages in DBD—has important implications. Wu et al. demonstrated that targeting mitochondrial complex I of CD177+ neutrophils alleviates lung IRI [35], and our finding that neutrophil influx is DCD-dominant suggests this strategy may be particularly relevant for DCD lungs. Sabashnikov et al. reviewed the role of neutrophil extracellular traps (NETs) in EVLP and their selective removal as a therapeutic strategy [36], which may be especially applicable to DCD lungs given our neutrophil dominance finding. Bonneau et al. correlated NET expression with PGD severity in human lung transplant recipients [24], and the DCD-dominant neutrophil pattern in our data suggests that NET-mediated injury may also be donor-type-dependent. Santos et al. reported that CCR5 drives NK cell-associated airway damage in lung IRI [37], and the DBD-dominant M1 macrophage pattern is consistent with brain-death-induced monocyte/macrophage pre-activation [8]. Farahnak et al. showed that B cells mediate lung IRI by recruiting classical monocytes [38], and our observation of DBD-dominant plasma cell expansion aligns with their findings. Kanou et al. demonstrated that cell-free DNA (cfDNA) levels in EVLP perfusate reflect tissue injury and can serve as biomarkers [39]; given the divergent injury mechanisms we identified, cfDNA profiles may also differ by donor type, warranting further investigation. Watanabe et al. showed that donor IL-17 receptor A regulates LPS-potentiated acute and chronic murine lung allograft rejection [40], highlighting the donor-side immune factors that may contribute to donor-type-specific outcomes.

### 4.4. Clinical Translational Implications

The donor-type-stratified PGD prediction models represent a paradigm shift from the current “one-size-fits-all” approach. The DCD model (AUC = 0.85) and DBD model (AUC = 0.83) both significantly outperformed the universal model (AUC = 0.76), and the cross-application analysis confirmed that each model is specifically effective for its corresponding donor type. Clausen et al. reviewed current knowledge on PGD [7], and our donor-type-stratified models add a new dimension to PGD risk assessment. This has direct clinical implications: PGD risk assessment should incorporate donor-type information, and prediction models should be donor-type-specific.

The proposed donor-type-aware EVLP protocols are particularly actionable. Gilmour et al. demonstrated that targeting sphingosine-1-phosphate receptor 1 protects pulmonary vascular endothelial integrity during human EVLP [41], and Premachandra et al. showed that EVLP can reverse warm ischemia-induced transcriptional changes [11]. Schiavon et al. provided a consensus statement on the use of normothermic perfusion machines in lung transplantation [42], establishing the clinical framework for protocol customization. Orlandi et al. demonstrated that preservation at 10◦C may better maintain cellular integrity and extend preservation time [43], offering an alternative preservation temperature that could differentially benefit DCD and DBD lungs. Cui et al. compared outcomes of DCD lung transplantation in the United States across procurement strategies incorporating direct procurement, normothermic regional perfusion and EVLP, and found outcome differences [44], highlighting the importance of procurement strategy as an additional layer of donor-type-specific optimization. Williams et al. reported on the use of normothermic regional perfusion in DCD donors for lung transplantation in the United States [45], noting that NRP-DCD lung allograft use remains underexplored. Thomassen et al. showed that twenty-four-hour hypothermic oxygenated perfusion preserves graft viability across both donation after circulatory death and donation after brain death [46], and our finding that DCD and DBD lungs have different drug sensitivity profiles suggests that EVLP solution additives should be customized: ferrostatin-1 and necrostatin-1 for DCD lungs (targeting metabolic collapse-driven ferroptosis/necroptosis), and baricitinib and VX-765 for DBD lungs (targeting inflammation-driven pyroptosis/apoptosis). Tanaka et al. categorized post-transplant outcomes of DCD donor lungs by donation after brain death extended criteria [47], providing a framework for integrating our donor-type-specific signatures with existing clinical criteria. Pergolizzi et al. reviewed strategies for maximizing lung utilization in both DBD and DCD donors [48], and our donor-type-aware approach directly supports this goal by enabling evidence-based donor-specific optimization.

### 4.5. Interpretive Caveats of the Donor-Type Dichotomy in Complex Clinical Practice

The binary DCD/DBD framework should be interpreted against the biological continuum of modern procurement practice. Mechanistic overlap arises predominantly in extended-criteria scenarios: DBD grafts may sustain secondary hemodynamic instability or unexpected arrest before recovery, whereas controlled-DCD lungs span wide agonal-phase ranges and increasingly undergo normothermic regional perfusion (NRP) that attenuates the very warm ischemia burden used to define them [44]. Neurogenic-inflammatory priming itself is heterogeneous—modulated by catecholamine exposure and hormonal resuscitation—so mixed-profile cohorts can blur donor-type contrasts and bias signatures toward procurement-intensity effects. A practical remedy is a two-dimensional framing—(warm ischemia duration/intensity) × (degree of neurogenic-inflammatory priming)—for positioning individual grafts along continuous axes rather than discrete classes. Consistent with this view, Allen et al. reported that transcriptional and cytokine differences between 97 DCD and 122 DBD lungs largely equalized after four hours of EVLP, implying a preconditioning effect that partially converges baseline divergence [49]. Donor-type signals should, therefore, individualize perfusion-drug strategies without excluding intermediate phenotypes, and the stratified models proposed here require prospective recalibration on mixed-profile populations.

### 4.6. Limitations

This study has several limitations:

(1) GSE18995 provides modest discovery depth (n = 35 factorial-complete samples). Although the 2 × 2 design affords high efficiency for interaction detection and effect directions replicated directionally in GSE127003/GSE268041, subgroup estimates still warrant cautious reading; permutation-based FDR control mitigates, but cannot eliminate, false discoveries at this scale.

(2) The discovery platform dates to 2009 (HG-U133 Plus 2.0 arrays); bulk transcriptomics lacks single-cell resolution, so low-abundance lymphocyte transitions may be underestimated despite LM22-constrained deconvolution, and cross-cohort confirmation reflects matched-platform subsets rather than fresh-generation RNA sequencing.

(3) PGD outcome datasets (GSE9102, GSE8021) provide incomplete donor-type metadata; where annotation was absent, a graded donor-type score derived from DCD/DBD signature genes served as proxy labeling, which introduces assignment noise that may dilute apparent stratified performance even though cross-application testing preserved the ranking pattern.

(4) All computational findings require prospective validation in clinical cohorts, and the proposed EVLP protocol adaptations need ex vivo dose–response testing and animal model verification before bedside translation.

(5) Interaction findings are associational: causal attribution between prioritized axes (e.g., RIPK3-driven necroptosis versus CASP1-driven pyroptosis) requires perturbation experiments such as chemical inhibition in precision-cut lung slices.

(6) External outcome validation relies on one mixed-donor cohort; generalization to contemporary NRP or hypothermic preservation pathways remains unassessed until prospectively designed registries capture those modalities.

(7) Contributing centers span heterogeneous procurement logistics—cold ischemia definitions, flush solutions, and storage temperatures—so residual biological heterogeneity can persist beyond statistically modeled batch effects.

(8) CMap connectivity profiles derive chiefly from non-transplant cell-line perturbations; candidate rankings inform hypothesis generation, whereas pharmacokinetics and dosing within EVLP circuits require dedicated work.

(9) Machine learning overfitting: Model development, feature pre-selection, and threshold tuning all occurred within the same five-cohort universe, so the stratified PGD signatures could encode cohort-specific idiosyncrasies—platform artifacts or center effects—rather than generalizable donor-type biology; regularization and donor-stratified cross-validation mitigate but cannot eliminate this risk.

(10) Optimism bias: The reported AUCs of the stratified models are apparent (in-sample) estimates and are expected to shrink in truly independent, prospectively collected cohorts; we, therefore, present them as hypothesis-generating benchmarks, and penalized refitting with bootstrapped optimism correction on larger multicenter cohorts should precede any clinical implementation.

## 5. Conclusions

This study demonstrates for the first time that DCD and DBD lungs undergo molecularly distinct IRI processes: DCD lungs experience metabolic collapse-driven ferroptosis and necroptosis, while DBD lungs experience inflammatory priming-driven pyroptosis and apoptosis. The 623 DCD-specific and 487 DBD-specific IRI genes, divergent metabolic and immune profiles, and donor-type-stratified PGD prediction models provide a new framework for precision lung transplantation. Donor-type-aware EVLP protocols represent an immediately translatable clinical strategy that could improve outcomes for both DCD and DBD lung recipients.

## Figures and Tables

**Table 1:** Summary of GEO datasets included in the study.

GEO ID	Phase	Platform	Deposited n	QC-Passed n	Final Analyzed n	Role
GSE18995	IRI	Affymetrix	42	42	35	Core discovery (2 × 2 factorial)
GSE127003	IRI	Affymetrix	92	92	92	DBD validation
GSE268041	IRI	Affymetrix	113	113	108	Mixed-donor validation
GSE9102	PGD	Affymetrix	30	30	26	PGD outcome (set 1)
GSE8021	PGD	Affymetrix	57	57	50	PGD outcome (set 2)

## Data Availability

All raw data were obtained from the NCBI GEO database (GSE18995, GSE127003, GSE268041, GSE9102, GSE8021). Result is available in the Supplementary Materials.

## Supplementary Materials

The following supporting information can be downloaded at: https://www.mdpi.com/article/10.3390/genes17091127/s1. Table S1: Dataset summary of the five GEO cohorts. Table S2A–C: donor-type interaction-effect genes, four-quadrant classification, and pathway-category summary. Table S3A–C: pathway enrichment, GSEA metabolic analysis, and ssGSEA scores. Table S4A,B: PCD pathway scores and PCD key genes. Table S5: CIBERSORTx immune deconvolution. Table S6A,B: WGCNA modules and hub genes. Table S7: PGD model comparison. Table S8: feature pool. Table S9: PGD signature genes. Table S10: CMap drug-repurposing results.

## Abbreviations

AUC	Area Under the Receiver Operating Characteristic Curve
CIT	Cold Ischemia Time
CIBERSORTx	Cell-Type Identification By Estimating Relative Subsets Of RNA Transcripts
CMap	Connectivity Map
DBD	Donation after Brain Death
DCD	Donation after Circulatory Death
EVLP	Ex Vivo Lung Perfusion
GEO	Gene Expression Omnibus
GSEA	Gene Set Enrichment Analysis
IRI	Ischemia–Reperfusion Injury
LASSO	Least Absolute Shrinkage and Selection Operator
LM22	Leukocyte Microarray signature with 22 immune cell subsets
NES	Normalized Enrichment Score
OXPHOS	Oxidative Phosphorylation
PCA	Principal Component Analysis
PCD	Programmed Cell Death
PGD	Primary Graft Dysfunction
RF	Random Forest
SHAP	SHapley Additive exPlanations
ssGSEA	Single Sample Gene Set Enrichment Analysis
WGCNA	Weighted Gene Co-expression Network Analysis
WIT	Warm Ischemia Time
XGBoost	Extreme Gradient Boosting
